# Population based absolute and relative survival to 1 year of people with diabetes following a myocardial infarction: A cohort study using hospital admissions data

**DOI:** 10.1186/1471-2458-10-338

**Published:** 2010-06-14

**Authors:** Sinead Brophy, Roxanne Cooksey, Michael B Gravenor, Clive Weston, Steven M Macey, Gareth John, Rhys Williams, Ronan A Lyons

**Affiliations:** 1 School of Medicine, Swansea University, SA2 8PP, UK; 2 Health Solutions Wales. Brunel House, Cardiff, CF24 0HA, UK

## Abstract

**Background:**

People with diabetes who experience an acute myocardial infarction (AMI) have a higher risk of death and recurrence of AMI. This study was commissioned by the Department for Transport to develop survival tables for people with diabetes following an AMI in order to inform vehicle licensing.

**Methods:**

A cohort study using data obtained from national hospital admission datasets for England and Wales was carried out selecting all patients attending hospital with an MI for 2003-2006 (inclusion criteria: aged 30+ years, hospital admission for MI (defined using ICD 10 code I21-I22). STATA was used to create survival tables and factors associated with survival were examined using Cox regression.

**Results:**

Of 157,142 people with an MI in England and Wales between 2003-2006, the relative risk of death or recurrence of MI for those with diabetes (n = 30,407) in the first 90 days was 1.3 (95%CI: 1.26-1.33) crude rates and 1.16 (95%CI: 1.1-1.2) when controlling for age, gender, heart failure and surgery for MI) compared with those without diabetes (n = 129,960). At 91-365 days post AMI the risk was 1.7 (95% CI 1.6-1.8) crude and 1.50 (95%CI: 1.4-1.6) adjusted. The relative risk of death or re-infarction was higher at younger ages for those with diabetes and directly after the AMI (Relative risk; RR: 62.1 for those with diabetes and 28.2 for those without diabetes aged 40-49 [compared with population risk]).

**Conclusions:**

This is the first study to provide population based tables of age stratified risk of re-infarction or death for people with diabetes compared with those without diabetes. These tables can be used for giving advice to patients, developing a baseline to compare intervention studies or developing license or health insurance guidelines.

## Background

Patients with diabetes who have an acute myocardial infarction (AMI) are at higher risk of death, both in the acute phase [1] and during follow-up [2-4]. In fact, cardiovascular disease is reported to account for almost 80% of all 'diabetic' deaths[5]. This may be because patients with diabetes often have a cluster of risk factors including hypertension, hypercholesterolemia, obesity, hyperglycemia, high risk lifestyle factors (such as smoking), albuminuria, and family history of disease [6] and have a more complications in hospital following an MI [7]. For example, heart failure is twice as common in patients with type 2 diabetes [8] compared with those without type 2 diabetes. Thus, the short term mortality rate of a patient with diabetes is reported to be twice that in those without diabetes [9,10] and patients with diabetes have reduced long term survival [2,11]. The relative risk of a primary MI is more common among women with type 2 diabetes than among men [12] However, there are currently few population-based estimates of the probability of survival following an AMI for people with diabetes compared with those without diabetes [2,13] and none look at time to re-infarction.

This study was developed from a project commissioned by the UK Department for Transport [14] to develop absolute and relative risk survival tables to show time to death or recurrence of an AMI for diabetic and non-diabetic patients hospitalised for AMI. It presents absolute risk reference tables for people who experience an AMI stratified by age, sex and diabetes and separately absolute risk reference tables for controls who have not experienced a primary AMI.

## Methods

### Design

We conducted a cohort study of risk of death or recurrence of MI in people hospitalised for MI using all hospital admissions in England and Wales for the years 2003-2006.

### Datasets

Data were obtained from two hospital admission datasets; PEDW - Patient Episode Database for Wales and HES - Hospital Episode Statistics for England. These datasets are publicly available on request to the data custodians. MI was defined using ICD code I21-I22 and diabetes using ICD 10 codes E10-E14. HES and PEDW records are held at a 'finished consultant episode' (FCE) level, which represent periods of care under a particular consultant while in hospital. One admission might therefore, result in more than one FCE. These FCE's were internally linked to develop 'hospital spells' for an individual and 'super-spells' that link hospital spells for patients transferred between hospitals during their index admission. Super-spells are records which give the entire patient journey through the hospital system. Data contained on the hospital admission systems includes demographics, diagnosis, surgery, admission and discharge information but it does not include prescriptions, symptoms or laboratory or other tests.

### Study population

Patients admitted to hospital who were aged less than 30 years were excluded from the study. Diabetes was defined as ICD codes E10-E14 (Insulin dependent, non-insulin dependent, malnutrition-related diabetes mellitus, other specified diabetes, and other non-specified diabetes). Surgery for the current MI was recorded (OPCS codes K40 to K50 and K75) which includes angioplasty, CABG and stent placement. All patients were taken from admission to hospital for an AMI between the dates 2003-2006.

### Linkage

An anonymised version of the PEDW database was linked with the Welsh NHS Administrative Register (AR) which records date of death or date of moving out of Wales. Therefore, an accurate estimation of death, or loss to follow-up due to leaving Wales, can be calculated. The HES dataset was linked with individual records of mortality held by the Office for National Statistics (ONS) using unique National Health Service (NHS) numbers [14]. Recurrent AMIs can be identified from readmissions to hospital. Patients who suffer AMI and die before reaching hospital cannot be identified by hospital-based systems, but can be identified using mortality data from AR and ONS. While there may be an underestimate of the number of re-infarctions the combined endpoint of re-infarction/death will include MI's resulting in death (without hospital admission). In the HES system patients who have moved out of England cannot be identified. This could have the effect of making the incidence of re-infarction and death slightly lower, since patients moving outside the UK who have an AMI or die cannot be identified. However, the number of people who moved out of England are relatively small and therefore unlikely to change the validity of the conclusions. In addition, patients who have a silent AMI, that is have a re-infarction but are not admitted and do not die, will be missed from this study.

### Control group

The entire Welsh population was selected using people on the Welsh AR and those who were not recorded as having had an MI before the 1^st ^of January 2003 were extracted. The rate of AMI and death in this control group was determined using the same methods as those for the AMI patients. The control group provided population-based incidence rates of first AMI or mortality in those without an index AMI since 1996.

### Quality of the datasets

Data from the Myocardial Ischaemia National Audit Project (MINAP) data [15] was obtained, with permission from the data guardians, for the period 1st April 2003-31^st ^March 2006 in order to examine validity of data held in the hospital admissions dataset. MINAP is a dataset of patients hospitalised with AMI which is used to compare the management of myocardial infarction within hospitals against targets specified by the National Service Framework for Coronary Heart Disease (NSF). This dataset is not a population register of AMI as it is known that participation and recruitment differs by hospital (for example percentage of non-STEMI MI's varies between hospitals from 0.8%-73%)[14]. The original reason for setting up MINAP was the measurement of the process and outcomes of the care of patients with MIs amenable to reperfusion treatment. Non-STEMI is not managed by reperfusion therapy and so some hospitals have tended not to record data for this type of heart attack. Consequently, not all MIs are included in MINAP. However, we have used this database to examine if all MI's found in MINAP can be also found in PEDW. This dataset was linked with the Welsh hospital admission system (PEDW) using anonymised and encrypted NHS numbers to examine the extent of overlap, coding coherence and accuracy.

In addition, the out-patients clinical care system used by the diabetologists was linked with the hospital admissions system to examine the number of known diabetic patients (as identified from the diabetes clinical care system) who had any mention of diabetes in their admission to hospital.

### Statistical analysis

STATA was used with the st command to examine survival (death alone or death and re-infarction) for patients stratified by age, sex and diabetes. Survival was analysed for the first 90 days, 91-180 days and 181-365 days. Factors associated with survival (diabetes, heart failure, age, gender and surgery [angioplasty/stenting, for the current MI]) were analysed using Cox regression. Interaction terms have not been included in the model as given the large numbers of patients all interaction terms are found to be significant and make interpretation of the model very difficult. Proportionality was examined by comparing graphs of the scaled Schoenfeld residuals.

## Results

### Data quality

In Wales, of 3,371 individuals recorded in MINAP attending Welsh hospitals, 3368 (99.91%) were also found in PEDW, 2,987 (89%) were found in PEDW with a diagnosis of AMI, 366 (10.9%) were recorded in PEDW but with a non AMI diagnosis (e.g. diagnosed with chronic ischemic heart disease or angina pectoris), 3.9% [15] were recorded as attending an English hospital with AMI and 3 (0.1%) were recorded in MINAP but missing from PEDW altogether.

There was no significant difference between the number of people recorded in MINAP as having diabetes (1620/8143 - 19.9%) compared with the number recorded on the hospital admission system, PEDW (30,407/160,367 - 20%). Thus, there is no evidence of relative under ascertainment of diabetes when using the hospital admission systems. In addition, of 853 patients who had diabetes (as confirmed on the out-patients diabetologist clinical care system) and who had an in-patient hospital visit (either related or not related to the diabetes), 774 (90%) had diabetes recorded during their hospital visit (for MI, cataract surgery, amputation).

Finally, in Wales, the number of people who moved outside of Wales in a 1 year period was 1.2%. We would expect the numbers moving out of the UK to be of similar levels[16]. Thus, the error level in the patients from the England system is likely to be small.

### Demographics

There were 30,407 (19%) people with diabetes (37.9% female, average age 70 (min-max: 30-120) and 129,960 (81%) people (35% female, average age 68.7 (min-max: 30-105) without diabetes. Among those with diabetes; 34.3% had heart failure and 15.3% had heart surgery for the MI. Among those without diabetes 20.6% had heart failure and 16.2% had heart surgery.

### Survival

**Figure 1:**
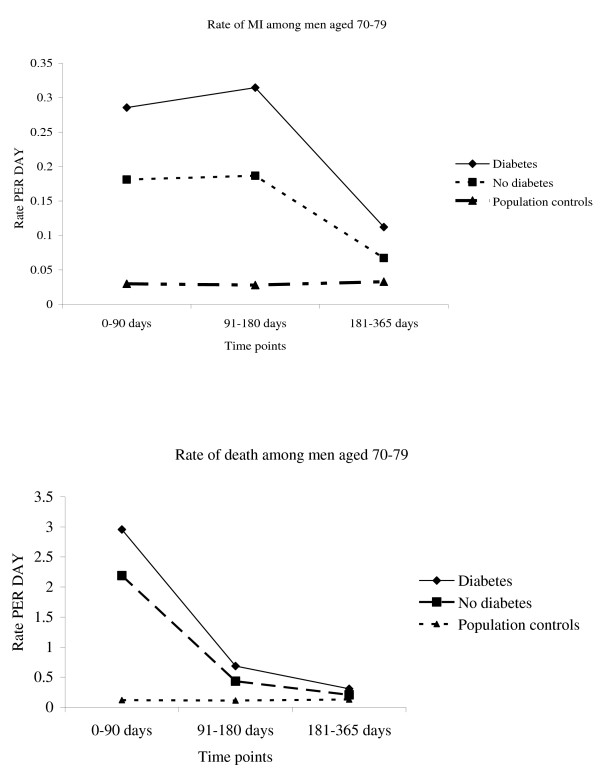
**Rate of MI or death with time. (Males aged 70-79)**.

There were 157,142 people followed for 126,731 follow-up years within England and Wales. An additional 3225 were included in the dataset but had no follow-up time as they died on the date of admission (2%), 8 patients were not followed as they were recorded as having moved out of Wales/UK and before the date of their admission for their MI. Within this timeframe there were 48,517 deaths or re-infarction events, giving an incidence of 38.3% (95%CI: 37.9-38.6) per person year of follow-up. The risk of death was highest in the first 90 days [Table 1], and then declined. However, the risk of re-infarction remained high for the first 180 days (Figure 1). The combined rate of death or re-infarction was highest for those with diabetes [Table 2]. Patients with diabetes were at 1.16 (95%CI: :1.1-1.2) times higher risk of death or re-infarction in the first 90 days, and at 1.5 (95%CI: 1.4-1.6) times higher risk at 90-365 days post MI, when controlling for age, gender, heart failure and heart surgery [Table 3]. Therefore, the attributable risk of the diabetes increases 90 days post MI. However, this is because the absolute risk of death in the non diabetic group declines greatly with time (see Figure 1).

**Table 1:** Mortality rates following an AMI per 1,000 people

Gender and Co-morbidity	Age	0-90 days: rate (95% CI)	n	91-180 days: rate (95% CI)	n	181-365 days: rate (95% CI)	n
**Male**							
No diabetes	30-39	13 (9-20)	1,635	1.5 (0.3-6.0)	1414	2.1(0.8-5.6)	1226
	40-49	17 (14-20)	7,894	3.5 (2.3-5.3)	6784	3.6 (2.5-5.1)	5722
	50-59	33 (30-36)	15,712	4.5 (3.5-5.9)	13276	4.3 (3.5-5.4)	11306
	60-69	77 (73-82)	18,701	15 (12-17)	15204	14 (12-16)	12761
	70-79	197 (190-203)	19,714	39 (36-42)	14224	37 (34-40)	11770
	80-89	395(383-407)	14,732	85 (79-92)	8904	91 (85-97)	6989
	90+	705 (665-749)	2,548	170 (140-190)	1192	160 (140-190)	870
Diabetes	30-39	19 (6-59)	170	15 (3.6-58)	150	5.3 (0.7-37.9)	123
	40-49	36 (25-51)	987	5 (2-14)	811	11 (5.9-19)	701
	50-59	67 (57-79)	2626	15 (11-22)	2120	14(10-19)	1808
	60-69	135 (124-147)	4496	25 (20-32)	3433	32 (27-38)	2875
	70-79	266 (251-280)	6133	62 (54-70)	4175	55 (49-62)	3374
	80-89	416 (392-442.3)	3528	130 (110-140)	2074	110 (95-120)	1529
	90+	704(604-817)	402	250 (180-340)	190	160 (110-230)	124
**Female**							
No diabetes	30-39	25 (13-50)	344	3.7 (0.5-26.3)	293	2.6 (0.3-18.6)	252
	40-49	28 (21-38)	1593	6.4 (3.2-12.9)	1341	4.7 (2.3-9.3)	1130
	50-59	51 (44-59)	3754	8.4 (5.6-12.6)	3117	6.2 (4.2-9.3)	2620
	60-69	88 (81-96)	7131	14(11-17)	5690	16 (14-20)	4795
	70-79	216 (207-225)	13298	38 (34-42)	9457	34 (31-38)	7747
	80-89	403 (391-415)	16908	78 (72-84)	10160	73 (69-79)	8074
	90+	629.9 (604-656)	5682	140 (120-150)	2853	130 (110-140)	2179
Diabetes	30-39	88 (37-212)	61	0 (0-0)	53	45 (15-140)	46
	40-49	73 (47-115)	290	28 (13-62)	235	17 (7.2-41)	193
	50-59	94 (74-119)	830	31 (20-49)	668	26 (17-39)	559
	60-69	172 (153-193)	2081	30 (22-40)	1532	32 (25-41)	1270
	70-79	277 (259-296)	4093	58 (49-68)	2749	60 (52-69)	2201
	80-89	419 (396-444)	3848	100 (91-120)	2240	110 (93-120)	1714
	90+	665 (600-738)	848	190 (150-240)	414	180 (140-220)	288

**Table 2:** Mortality and re-infarction rate following a AMI per 1,000 people

Gender and Co-morbidity	Age	0-90 days: rate (95% CI)	n	91-180 days: rate (95% CI)	n	181-365 days: rate (95% CI)	n
**Male**							
No diabetes	30-39	20 (14-28)	1635	9.1 (5.2-16)	1414	4.8 (2.5-9.2)	1226
	40-49	24 (20-28)	7894	9.5 (7.3-12.2)	6784	8.9 (7.1-11.1)	5722
	50-59	43 (40-46)	15712	11 (9.5-13)	13276	8.6 (7.3-10.1)	11306
	60-69	88 (84-93)	18701	24 (21-27)	15204	20 (18-22)	12761
	70-79	213 (206-221)	19714	56 (52-60)	14224	49 (46-52)	11770
	80-89	421 (409-434)	14732	113 (106-121)	8904	110 (100-120)	6989
	90+	735 (693-779)	2548	202 (176-232)	1192	190 (170-220)	870
Diabetes	30-39	19 (6-59)	170	29 (11-78)	150	5.34 (0.75-38)	123
	40-49	53 (40-70)	987	12 (6-23)	811	24 (16-36)	701
	50-59	87 (76-100)	2626	28 (21-36)	2120	22 (17-29)	1808
	60-69	154 (142-167)	4496	47 (40-55)	3433	46 (40-53)	2875
	70-79	292 (277-307)	6133	90 (81-100)	4175	75 (68-84)	3374
	80-89	453 (427-480)	3528	174 (156-195)	2074	150 (140-170)	1529
	90+	701 (634-776)	402	301 (226-402)	190	190 (130-270)	124
**Female**							
No diabetes	30-39	25 (13-50)	344	11 (3.6-35)	293	5.2 (1.3-21)	252
	40-49	38 (29-49)	1593	14 (8.6-22)	1341	9.9 (6.1-16)	1130
	50-59	60 (51-68)	3754	13 (12-21)	3117	12 (9-16)	2620
	60-69	100 (93-108)	7131	25 (21-29)	5690	21 (18-25)	4795
	70-79	232 (223-241)	13298	54 (49-59)	9457	46 (42-50)	7747
	80-89	424 (412-435)	16908	103 (97-110)	10160	93 (87-98)	8074
	90+	646 (620-673)	5682	165 (150-182)	2853	140 (130-160)	2179
Diabetes	30-39	88 (37-212)	61	21 (2.9-148)	53	60 (23-160)	46
	40-49	100 (68-147)	290	51 (28-93)	235	38 (21-69)	193
	50-59	99 (79-125)	830	47 (33-68)	668	34 (24-48)	559
	60-69	188 (168-210)	2081	60 (48-74)	1532	49 (40-60)	1270
	70-79	301 (283-321)	4093	97 (86-110)	2749	81 (72-92)	2201
	80-89	459 (433-484)	3848	144 (128-162)	2240	130 (120-150)	1714
	90+	701 (634-776)	848	227 (182-283)	414	200 (160-250)	288

**Table 3:** Cox regression analysis for risk of re-MI or death

Time from initial AMI	Risk factor	Crude Hazard Ratio (95%CI)	Adjusted Hazard Ratio (95%CI)
0-90 days	Diabetes	1.3 (1.26-1.33)	1.16 (1.12-1.19)
	Heart failure	2.6 (2.5-2.6)	1.6 (1.6-1.69)
	Age group (per increase in decade)	1.06 (1.06-1.06)	1.04 (1.04-1.05)
	Heart surgery for current MI	0.16 (0.15-0.17)	0.28 (0.27-0.3)
	Female gender	1.6 (1.56-1.63)	1.0 (0.98-1.03)
91-180 days	Diabetes	1.71 (1.6-1.8)	1.5 (1.4-1.6)
	Heart failure	3.1 (2.96-3.2)	1.96 (1.86-2.1)
	Age group (per increase in decade)	1.06 (1.06-1.07)	1.05 (1.04-1.05)
	Heart surgery for current MI	0.24 (0.21-0.26)	0.42 (0.37-0.46)
	Female gender	1.52 (1.4-1.6)	0.94 (0.9-1.0)
180-365 days	Diabetes	1.66 (1.57-1.75)	1.5 (1.4-1.5)
	Heart failure	3.06 (2.9-3.2)	1.93 (1.8-2.03)
	Age group (per increase in decade)	1.066 (1.063-1.068)	1.055 (1.05-1.06)
	Heart surgery for current MI	0.299 (0.27-0.32)	0.52 (0.47-0.57)
	Female gender	1.5 (1.4-1.6)	0.94 (0.9-0.99)

The population risk is given in Table 4 and the relative risk compared with population rates is given in Table 5. The risk attributable to having an AMI is highest in younger ages and directly after the MI (relative risk of death or re-infarction is 82.3 among women aged 40-49 with no diabetes at 0-90 days compared with 1.1 for women aged 90 at 181-365 days. The relative risk for those with diabetes is highest for younger patients. For example, there is a 2 fold higher risk of death or re-infarction for males with diabetes aged 50-59 at 0-90 days post MI (compared with those without diabetes, 38/18.6 = 2.0 (95%CI: 1.6-2.7) higher risk -Table 5). However, in males aged 90+ there is no increased risk associated with having diabetes. (12.5/11.9 = 1.05 (95%CI: 0.82-1.9) at 0-90 days. Thus, the relative risk of mortality/re-infarction is more prominent in younger individuals with diabetes because the risk in controls is so much lower at a younger age. Age is a very strong determinant of both death and myocardial infarction as the absolute risk of these outcomes almost doubles with each age decade (Table 1 and 2).

**Table 4:** Rate of morality or infarction in controls (with no previous history of AMI) per 1,000 people

Gender	Age	0-90 days: rate	n	91-180 days: rate	n	181-365 days: rate	n
**Male**							
	30-39	0.46	226,251	0.38	225,283	0.71	224,530
	40-49	0.85	210,842	0.78	210,255	1.8	209,697
	50-59	2.3	202,353	2.16	201,602	4.7	200,923
	60-69	4.9	149,269	5.06	148,381	11.7	147,462
	70-79	13.3	102,893	12.45	101,414	29.07	100,052
	80-89	30.0	40,259	26.99	39,019	68.78	37,922
	90+	58.7	5,061	52.64	4,758	125.55	4,487
Female	30-39	0.17	219,185	0.17	218,507	0.3942	217,754
	40-49	0.46	203,768	0.57	203,322	1.05	202,860
	50-59	1.40	199,492	1.13	198,944	2.39	198,432
	60-69	3.02	153,572	2.93	152,927	6.88	152,294
	70-79	8.42	127,186	8.22	125,988	19.44	124,827
	80-89	21.96	75,909	21.37	74,142	52.52	72,464
	90+	54.88	16,421	50.26	15,505	128.61	14,718

**Table 5:** Relative risk of death or MI post hospitalization for MI

		0-90 days (95%CI)	91-180 days (95%CI)	181-365 days (95%CI)			0-90 days (95%CI)	91-180 days (95%CI)	181-365 days (95%CI)
**Male**					**Female**				
No diabetes	30-39	43.1 (29.1-63.7)	23.9 (13.3-42.9)	6.76 (3.0-15.3)	No diabetes	30-39	148.6 (71.5-308.5)	64.3 (20.7-200)	13.1 (2.3-73.6)
	40-49	28.3 (23.1-34.7)	12.2 (9.1-16.2)	4.9 (3.7-6.6)		40-49	82.3 (59.8-113.3)	24.2 (14.9-39.4)	9.4 (5.2-17.1)
	50-59	18.6 (16.5-20.9)	5.2 (4.3-6.2)	1.8 (1.5-2.2)		50-59	42.8 (36.1-50.9)	14.0 (10.2-19)	5.0 (3.5-7.2)
	60-69	18.0 (16.5-19.6)	4.7 (4.2-5.3)	1.6 (1.5-1.9)		60-69	33.1 (29.6-37.2)	8.3 (6.9-10.1)	3.1 (2.5-3.8)
	70-79	16.1 (15.1-17.0)	4.5 (4.1-4.9)	1.7 (1.5-1.8)		70-79	27.5 (25.7-29.4)	6.5 (5.9-7.2)	2.3 (2.1-2.6)
	80-89	14.1 (13.2-14.9)	4.2 (3.8-4.6)	1.6 (1.5-1.7)		80-89	19.3 (18.3-20.3)	4.8 (4.4-5.2)	1.7 (1.6-1.9)
	90+	12.5 (11.2-19.6)	3.8 (3.2-4.5)	1.5 (1.3-1.8)		90+	11.8 (11-12.6)	3.2 (2.9-3.7)	1.1 (0.97-1.2)
Diabetes	30-39	41.0 (13.6-123.3)	75.4 (29-196.2)	7.5 (0.6-84)	Diabetes	30-39	518 (217-1235)	122 (18.7-798.8)	152 (47.6-487.3)
	40-49	62.1 (45.9-84.0)	15.2 (7.9-29)	13.5 (8.3-21.8)		40-49	218 (146-325)	90.7 (50.9-161.6)	35.9 (17.4-74.1)
	50-59	38.0 (32.6-44.2)	12.8 (9.8-16.7)	4.7 (3.5-6.5)		50-59	71 (56-90)	41.9 (29.0-60.3)	14.3 (9.1-22.3)
	60-69	31.4 (28.4-34.7)	9.2 (7.7-10.8)	3.9 (3.3-4.7)		60-69	62.2 (54.8-70.7)	21.03 (17.0-26.1)	7.1 (5.5-9.1)
	70-79	21.9 (20.6-23.4)	7.2 (6.4-8.1)	2.5 (2.3-2.9)		70-79	35.8 (33.2-38.6)	11.8 (10.4-13.5)	4.2 (3.6-4.8)
	80-89	15.1 (14.1-16.1)	6.4 (5.8-7.2)	2.1 (1.9-2.5)		80-89	20.9 (19.7-22.1)	6.74 (6.0-7.5)	2.5 (2.2-2.8)
	90+	11.9 (10.5-13.6)	5.7 (4.5-7.3)	1.5 (1.0-2.2)		90+	12.8 (11.8-13.8)	4.5 (3.7-5.4)	1.5 (1.2-2.0)

Although female gender carries a risk of 1.6 that of males in unadjusted analysis, after correction for age, this factor is no longer significant (Table 3).

## Discussion

This study presents population based figures for risks of recurrence of MI or death for 30,407 people with diabetes following an MI compared with those without diabetes. These tables can give the absolute risks and the relative risks of MI and/or death stratified by age and sex. Findings suggest that diabetes is an important risk factor for younger patients and is associated with poorer outcome especially for re-infarction. This is supported by the finding that many young people experiencing MI have undiagnosed diabetes but an absence of other risk factors [17]. Risk factors specifically associated with diabetes include diabetes increased atherosclerotic plaque formation and thrombosis [18] associated with hypergycemia [19].

The control population risk is constant with time (e.g. 0.46 per 1,000 in the age 30-39 over 0-90 days and 0.71 over 181-365 days (0.36 per 1,000 if this is taken as a 90 day period- Table 4). There are no existing studies showing survival to re-MI in a population based study [11]. Thus, this study provides survival tables that can be used to improve information regarding prognosis for patients, to inform treatment strategies for people having an AMI and to inform driver licensing and insurance guidelines. This is a population based study based on 30,407 people with diabetes. No other study has looked at a total population cohort to give rates of recurrence of infarction stratified by age and sex. This data is not possible to generate using meta-analysis of existing cohort studies [20] and can only be developed using routinely collected data.

Data on mortality are comparable to that previously published showing a 2 fold increase in mortality in the short term [9,10] and 40% increase in the longer term [2,13] for MI patients with and without diabetes.

However, using routine data does have its limitations. Coding and recording of all AMI's in the hospital admissions dataset is not perfect and there may be an underestimate of number of AMI's by approximately 10%, as some MI's are recorded using imprecise coding. In addition, approximately 10% of the 'non-diabetes' group may have had diabetes but the code was missing from the hospital admission data.

Calculating unbiased population risks is difficult due to absence of disease registers with 100% coverage and selection bias[20] and restricted inclusion criteria may lead to distorted risks. Therefore, routinely collected data, although imperfect, is currently the closest we have to a population based disease register which supports low cost analysis of up to date and current rates of disease and survival times for hospitalised MI. However, to fully understand the explanatory or confounding factors and to incorporate them into a multivariate cox regression analysis, linkage is needed with more data sources. For example factors which need to be incorporated would include; other co-morbidities (peripheral arterial disease, chronic pulmonary disease, hypertension, hyperlipidemia, chronic kidney disease and others), lifestyle factors (smoking, diet, physical activity, physiotherapy), treatments prescribed (beta blockers, streptokinase, aspirin, statins (pre and post MI)), location of infarction (anterior, inferior) and type of AMI (STEMI, non-STEMI). To improve survival models linkage will be needed with primary care datasets where the most complete source of morbidity data is held.

## Conclusions

In summary, this study provides population based age stratified absolute risks of re-infarction or death for people with diabetes. These tables are based on all hospital admissions in England and Wales for the period 2003-2006 and these absolute risks can be used as reference tables in clinical care, industry and in informing driving legislation.

## List of abbreviations

AMI: acute myocardial infarction; AR: Administrative Register; FEC: finished consultant episode; OPCS: Office of Population, Censuses and Surveys: Classification of Surgical Operations and Procedures; ONS: Office for National Statistics; HES: Hospital Episode Statistics for England; ICD: International Classification of Disease; MINAP: Myocardial Ischaemia National Audit Project; NHS: National Health Service; NSF: National Service Framework for Coronary Heart Disease; PEDW: Patient Episode Database for Wales; RR: relative risk; STEMI: ST segment elevation myocardial infarction

## Competing interests

The authors declare that they have no competing interests.

## Authors' contributions

The study was designed by SB, RL, CW, and RW. Data extraction was conducted by SM and GM. Analysis was undertaken by SB, RC, MG and RL. The first draft of the manuscript was undertaken by SB, RC and SM and comments and changes were made by MG, CW, GJ, RW and RL. All authors have approved the final manuscript.

## Pre-publication history

The pre-publication history for this paper can be accessed here:

http://www.biomedcentral.com/1471-2458/10/338/prepub
